# Comparison of Colorectal Cancer Stem Cells and Oxaliplatin-Resistant Cells Unveils Functional Similarities

**DOI:** 10.3390/cells11030511

**Published:** 2022-02-01

**Authors:** Vanessa Rodríguez-Fanjul, Rosa Guerrero-López, Beatriz Fernández-Varas, Rosario Perona, Ana Sastre-Perona, Leandro Sastre

**Affiliations:** 1 Instituto de Investigaciones Biomédicas CSIC/UAM and CIBER de Enfermedades Raras (CIBERER), 28029 Madrid, Spain; vanessa.rodriguez@imdea.org (V.R.-F.); rosaguerrero@iib.uam.es (R.G.-L.); bfvaras@iib.uam.es (B.F.-V.); rperona@ext.iib.uam.es (R.P.); 2 Biomarkers and Experimental Therapeutics in Cancer, IdiPAZ, 28046 Madrid, Spain

**Keywords:** colorectal cancer, CRC, cancer stem cells, cancer-initiating cells, oxaliplatin, drug resistance, tumor recurrence, chemotherapy

## Abstract

Colorectal cancer is the second most common cancer in women, the third in men, and an important cause of cancer-related mortality. Recurrence and the development of chemotherapy resistance are major hindrances for patients’ treatment. The presence of cancer stem cells with chemotherapy resistance able to generate proliferating tumor cells contributes to tumor recurrence and resistance. In addition, tumor cells can develop chemoresistance through adaptation mechanisms. In this article, cancer stem cells were isolated from HT29 and SW620 colorectal cancer cell lines. Oxaliplatin resistance was induced by a single drug treatment simulating the usual guidelines of patient treatment. A comparison of these two populations showed similarities since cancer stem cells presented increased oxaliplatin resistance, and resistant cells contained an increased number of cancer stem cells. Cancer stem cells isolated from resistant cells showed increased oxaliplatin resistance. Cell invasion capacity and epithelial-mesenchymal transition were increased both in cancer stem cells and oxaliplatin-resistant cells. mRNA expression analysis showed that both cell types shared a significant proportion of commonly regulated genes. In summary, the data presented indicate that colorectal cancer stem cells and oxaliplatin-resistant cells are highly related cell populations that might have interesting implications in the development of tumor recurrence and resistance to chemotherapy.

## 1. Introduction

Colorectal cancer (CRC) is the fifth most common type of cancer, with nearly 1.1 million cases diagnosed in 2020 around the world. It is the third most common cancer in men and the second in women. It represents the second leading cause of death by cancer [1,2]. The survival rate for patients with metastatic CRC is low: 24% to 36 months and 13% to five years [3]. CRC can be treated with surgical resection, radiation, and/or chemotherapy. Standard first- and second-line treatments of CRC are based on the combination of 5-fluorouracil plus oxaliplatin or irinotecan [4]. This combined treatment produces response rates of 40–50% in the patients [5]; however, many patients do not respond to chemotherapy, presenting a low overall survival [6].

Several mechanisms have been involved in CRC progression and chemotherapy resistance, including the presence of cancer stem cells (CSCs) and the process of epithelial-mesenchymal transition (EMT) [7,8]. Cancer stem cells constitute a small population of tumoral cells that harbor stem cell properties, such as the capacity of self-renewal and differentiation to other cells within the tumor. In CRC, they were initially described as a cell population capable of initiating tumor growth in immunodeficient mice [9,10]. In addition to their tumor-initiating capacity, CSCs are able to resist chemotherapy [11,12,13,14,15]. EMT is a program that drives morphogenetic changes in epithelial cells, characterized by the loss of cell–cell adhesion and cell polarity and by the acquisition of the migratory and invasive capacity of mesenchymal cells [16]. Therefore, EMT is crucial in tumor progression and metastasis. In addition, several studies have shown that EMT generates cells with CSC properties [17,18]. These data enforce the role of CSCs in cancer progression, invasion, and metastasis.

A large proportion of patients with advanced CRC tend to develop metastasis and chemoresistance [19]. This recurrence has been associated with the presence of CSCs because these cells are enriched following chemotherapy and radiation [20,21]. These CSC cells are proposed to proliferate asymmetrically and differentiate to re-populate the tumor. However, CSCs are a minority of cells within a tumor, and a relatively larger number of cells remain after chemotherapy [22]; several additional mechanisms have been proposed to take part in the development of resistant tumors. For example, Bose et al. proposed that chemoresistant CSCs secrete soluble factors that mediate the survival of the surrounding otherwise chemosensitive tumor cells [23]. Other authors have proposed that non-stem cancer cells can dynamically convert into the stem-like state [24]. CSC conversion has also been described in colon cancer cell lines [25]. This transition can be induced by tumor environment factors that promote EMT with a therapeutic resistant phenotype [17,26]. For example, the expression of the SNAIL transcription factor in CRC cells regulates the expression of CSC transcription factors [27]. In addition, integrin-like kinase (ILK) expression in CRC induces markers of EMT, CSCs, and chemoresistance [28]. Chemotherapy treatment has also been described to be associated with the CSC phenotype [29,30].

Studies in non-small cell lung cancer cell lines have shown that CSCs and cisplatin-resistant cells showed similar characteristics [31]. Hu et al. also described that the dedifferentiation of CRC cells contributes to chemotherapy resistance [32].

The studies summarized above enforce the importance of CSCs in CRC progression, metastasis, and chemoresistance, and some of them suggest the possible existence of the transition between differentiated and CSCs. These possible transition processes could be very relevant for the treatment of advanced and metastatic cancer, including CRC. The aim of the present study is to generate further knowledge about the possible relationship between CSCs and treatment resistance in CRC. Two different CRC cell lines were used: HT29 derived from a colorectal adenocarcinoma and SW620 derived from a lymph node metastasis of a colorectal adenocarcinoma. HT29 presents APC, BRAF, PIK3CA, and TP53 mutations, whereas SW620 cells present APC, KRAS, and TP53 mutations [33]. Both cell lines were made resistant to oxaliplatin, a drug frequently used in advanced CRC treatment [34,35]. CSCs were generated by culture in defined media under non-adherent conditions (3D culture) [36] and presented increased resistance to oxaliplatin. In complementary studies, oxaliplatin-resistant cells were generated by a single-dose treatment with oxaliplatin to mimic the usual therapeutic protocol. These resistant cells were shown to present CSCs characteristics, including the capacity to form tumor spheroids, increased migration, and expression of EMT markers. These data are in agreement with possible crosstalk between CSCs and oxaliplatin-resistant CRC cells.

## 2. Materials and Methods

### 2.1. Cell Culture and Selection of Oxaliplatin Resistant and CSC Populations

HT29 (ATCC HTB-38) and SW620 (ATCC CCL-227) human colorectal cancer cells were purchased from the American Type Culture Collection and maintained in DMEM/F12 media supplemented with 10% bovine fetal serum (FBS), 0.5 µg/mL fungizone, 40 µg/mL gentamicin, and 2 mM glutamine. A tridimensional (3D) culture of the cancer stem cell population was made in DMEM/F12 (1:1) supplemented with 2 mM glutamine, 5 mM HEPES, 0.4% BSA (Bovine Serum Albumin), N2 supplement (Gibco, Carlsbad, CA, USA), 20 ng/mL EGF and bFGF (Prepro-Tech, Cranbury, NJ, USA), 0.5 µg/mL fungizone, and 40 µg/mL gentamicin using ultra-low attachment plastic dishes, as previously described [31]. Oxaliplatin-resistant cells were obtained by culturing HT29 and SW620 cells with 160 µg/mL and 6 µg/mL, respectively, oxaliplatin for 72 h. After this treatment, cells were cultured in the absence of the drug. Cell cultures were maintained at 37 °C in a humidified 5% CO_2_ atmosphere.

### 2.2. Cell-Viability Assays

Cell viability was determined using the MTS hydrolysis method (Promega Corporation, Madison, WI, USA) following the manufacturer’s instructions. Briefly, cells were cultured in 96-well plates at a density of 50 cells/well for 24 h. Oxaliplatin was added at the concentrations indicated in each experiment, and the culture continued for 72 h. After that time, cell viability was determined by adding 20 µL of MTS, incubating for 3 h, and determining the absorbance to 490 nm. Each experiment was repeated at least three times using quadruplicate samples.

### 2.3. Clonogenicity Assays

The capacity of the cells to grow as clones from single cells was assayed in liquid culture by seeding a total of 48 cells in a 96-well plate. Cells were cultured for 10 days, and the number of clones formed was determined by microscopic observation. To determine the formation of spheroids, ultra-low attachment plates and a defined medium were used. Control experiments were performed using adherent plates and FBS-containing media. At least 10 experiments were conducted in duplicate to determine the average number of clones formed under adherent and non-adherent conditions. The proportion of CSC was calculated as a percentage of the number of spheroids grown in suspension in comparison to the number of clones grown in 2D.

### 2.4. In Vitro Cell Invasion Assays

Cell invasion assays were performed using BD BioCoat^TM^ Matrigel^TM^ invasion chambers (BD Biosciences, Palo Alto, CA, USA). First, 10^4^ cells were seeded in the upper chamber in a medium containing 0.5% FBS and 0.1% BSA. A culture medium containing 10% FBS was added to the lower part of the chamber. In the case of CSCs, the defined media used for the upper chamber contained 0.1% BSA without growth factors, while complete conditioned media was added to the lower chamber. After 24 h of culture, cells in the upper chamber were removed. Cells that invaded the lower part of the chamber were stained using the Diff Quick method (Medion Diagnostics, Miami, FL, USA) and quantified using the analysis program Soft Imaging Systems (Olympus, Münster, Germany). At least three experiments were conducted using triplicate samples.

### 2.5. Quantitative RT-PCR Analysis of Gene Expression

Total cellular RNA was isolated using the Trizol reagent (Invitrogen, Carlsbad, CA, USA) and purified with the RNeasy Mini Kit (Qiagen, Valencia, CA, USA). The High-Capacity cDNA archive kit (Applied Biosystems, Waltham, MA, USA) was used to covert 1 µg of each RNA into cDNA. Quantitative PCR was carried out using TaqMan probes and the Taq-man Universal PCR Master Mix (Applied Biosystems). The following probes were utilized: Hs00195591_m1 (SNAI1 gene), Hs00950344_m1 (SNAI2), Hs00232783_m1 (ZEB1), Hs00365052_m1 (FN1), Hs00242571_m1 (IFI6), Hs00923290M_1 (ADAM8), Hs00973637_m1 (OAS1), Hs01023894_m1 (CDH1, E-cadherin), Hs00983056_m1 (CDH2, N-cadherin), Hs00185584_m1 (Vimentin), and Hs03929097_g1 (GAPDH). CD133 mRNA levels were determined using the Power SYBR kit (Applied Biosystem) and the following oligonucleotides: CD133 (TCTCTATGTGGTACAGCCG and TGATCCGGGTTCTTACCTG) and GAPDH (GAGAGACCCTCACTCTG and GATGGTACATGACAAGGTGC). The StepOne Plus Real-Time PCR System (Applied Biosystems) was used for quantitative PCR. Relative expression levels were determined by the comparative threshold cycle method using GAPDH as an internal control.

### 2.6. Gene Expression Analysis by RNA Sequencing

RNA was isolated using Trizol reagent (Invitrogen, Carlsbad, CA, USA) and purified with the RNeasy Mini kit (Qiagen, Valencia, CA, USA). RNA quantity was determined using Qubit 2.0 (Life Technologies, Carlsbad, CA, USA), and the quality was assessed using the Bioanalyzer 2100 (Agilent Technologies, Santa Clara, CA, USA). mRNA purification, conversion to cDNA, DNA sequencing, and data analysis were carried out by the Sistemas Genomicos S.L. (Valencia, Spain). The sequencing of the libraries was conducted by the Paired-End (75 + 35 nt) method using a SOLID 5500xl system. Sequences were aligned to the human genome using Bioscope1.3 software (http://solidsoftwaretools.com, accessed on 6 November 2009). The program Cufflinks v2.02 [37] was used for transcript identification and quantification. For data normalization, the program EDASeq (http://www.bioconductor.org/packages/2.11/bioc/html/EDASeq.html, accessed on 6 November 2009) was used, and differential expression was calculated using DESeq [38] considering a fold change value higher than 1.5 or lower than −1.5 and a probability lower than 0.01. Genes differentially expressed in oxaliplatin-resistant cells and CSCs were compared using the Venny program (https://bioinfogp.cnb.csic.es/tools/venny/index.html, accessed on 02 December 2021).

### 2.7. Statistical Analysis of the Data

For the statistical analysis of the results, the mean was used to measure the main tendency of the data, and the standard deviation was used for dispersion measurement. All statistical analysis was performed using PRISM software (GraphPad Software, Inc., La Jolla, CA, USA). The differences between the mean values of each group were compared using Student’s *t*-test for two groups of samples and one-way analysis of variance (ANOVA) followed by Dunnet’s test for multiple comparisons. A value of *p* < 0.05 was considered statistically significant. The Graphical Abstract was created with BioRender.com.

## 3. Results

### 3.1. Culture of Colorectal Cancer Stem Cells

**Figure 1:**
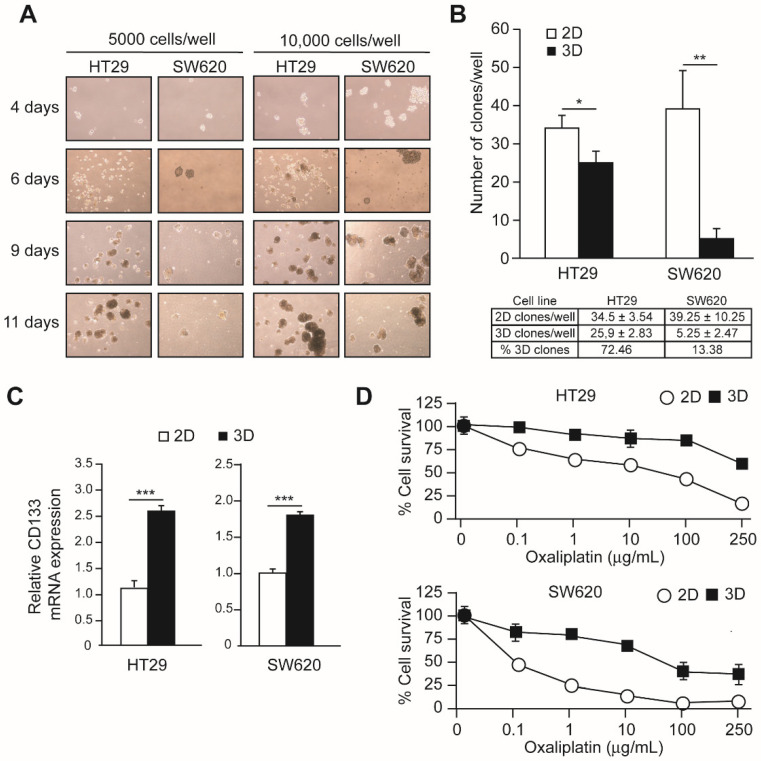
Characterization of 3D cultured colon CSCs and oxaliplatin sensitivity. Panel (**A**). Five thousand cells per well or ten thousand cells/well of the CRC cell lines HT29 or SW620 were cultured in non-adherent plates in defined media for 4, 6, 9, or 11 days, as indicated. Pictures were taken using a Nikon TS100 microscope. Panel (**B**). HT29 and SW620 cells were cultured in 96-well plates at an average density of 46 cells/plate for 10 days. In the case of 2D culture (open bars) adherent plates were used, while in 3D culture (black bars), non-adherent plates and defined media were used. The number of clones obtained is represented in the upper graphic and indicated in the lower table. Panel (**C**). RNA was isolated from HT 29 and SW620 cells grown under 2D (open bars) or 3D (black bars) and the amount of CD133-coding mRNA was determined by RT-qPCR. Panel (**D**). HT29 (left graph) and SW620 (right graph) cells were incubated in the presence of the indicated amounts of oxaliplatin for 3 days. The percentage of cell survival in relation to cells grown without oxaliplatin is represented for each concentration. Experiments were repeated three times with similar results. Statistics: * *p* < 0.05; ** *p* < 0.01; *** *p* < 0.001.

The human colon adenocarcinoma cell line HT29 and the colorectal adenocarcinoma cell line SW620 were cultured under non-adherent conditions in a defined, serum-free medium (3D culture) in order to select spheroid-forming cancer stem cells. Spheroids of similar sizes were obtained from both cell lines after 6 days of culture (Figure 1A). The spheroid-forming capacity of each cell line was determined by distributing an estimated number of 48 cells in a 96-well plate to assure single cells in each well and measuring the number of clones formed after 10 days of culture. Adherent plates were used as controls of the number of seeded cells. The numbers of clones obtained under adherent (2D) and non-adherent (3D) conditions are represented in Figure 1B. Spheroids obtained in 3D cultures were originated from single cells that were considered cancer stem cells (CSCs). The percentage of CSCs in the HT29 cell line was very high (72.46%) and much larger than that observed in SW620 cells (13.38%). The expression of the mRNA coding for the colorectal CSC marker CD133 was determined in cells grown under 2D and 3D conditions by RT-qPCR (Figure 1C) In both cell lines, cells cultured under 3D conditions showed significantly increased expression of CD133.

CSCs frequently show increased resistance to chemotherapy. To determine if this was the case in the present model, oxaliplatin sensitivity was determined. HT29 and SW620 cells were cultured for 15 days under 3D conditions, control cells were incubated with increasing amounts of oxaliplatin, and cell survival was determined after 3 days of treatment. The results obtained are shown in Figure 1D. HT29 and SW620 cells cultured under 3D conditions presented a marked decrease in oxaliplatin sensitivity.

### 3.2. Isolation of Oxaliplatin Resistant Cells

**Figure 2:**
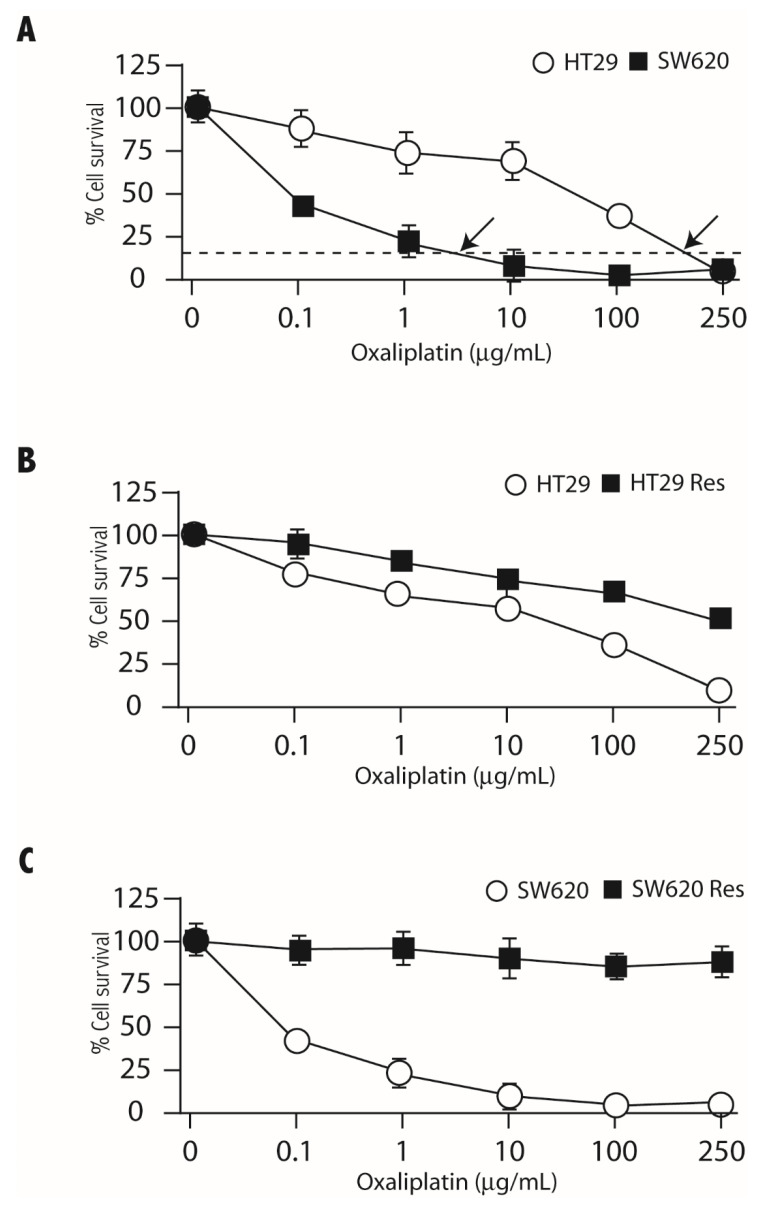
Generation of oxaliplatin-resistant CRC cells by a single treatment with the drug. Panel (**A**). HT29 (open circles) and SW620 (black squares) were incubated in the presence of the indicated concentrations of oxaliplatin for 3 days. The percentage of cell survival in relation to cells incubated without drug is indicated. Arrows indicate the concentrations corresponding to 20% of cell survival (IC80). Panels (**B**,**C**). HT29 (**B**) or SW620 (**C**) cells were cultured for three days with the oxaliplatin concentrations corresponding to the IC80 of the corresponding sensitivity curves. After this treatment, cells were cultured in the absence of the drug for 7 days, and the oxaliplatin sensitivity was determined as described in panel (**A**). Open circles represent the untreated cells, and black squares represent the oxaliplatin-resistant cells. Experiments were repeated three times with similar results.

Oxaliplatin-resistant cells were isolated by incubation with a single dose of the drug. Oxaliplatin sensitivity of HT29 and SW620 cells was determined in 2D culture to establish the drug concentration that decreased cell survival by 80% (IC80) (Figure 2A). These concentrations were 160 µg/mL for HT29 cells and 6 µg/mL for SW620 cells (indicated by a dotted line and arrows in Figure 2A). Oxaliplatin-resistant cells were obtained by culturing HT29 and SW620 cells in the presence of the IC80 concentration of the drug for three days. Oxaliplatin sensitivity of the cells that survived this single-dose treatment was determined, as shown in Figure 2B,C. Surviving cells showed decreased sensitivity to oxaliplatin in both cells lines and were considered resistant cells. Resistance was maintained after several weeks of culture and after freezing and re-culturing of the cells.

**Figure 3:**
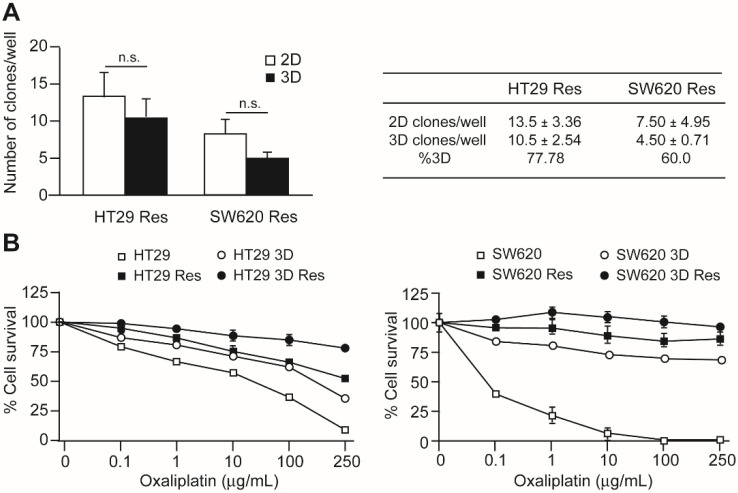
Clonogenic capacity and oxaliplatin sensitivity of oxaliplatin-resistant CRC cells. Panel (**A**). Oxaliplatin-resistant HT29 (HT29Res) and SW620 (SW620Res) cells were cultured in 96-well plates at a density of 46 cells/plate for 10 days. Control cells were cultured in adherent plates (2D, open squares), while CSCs were cultured in non-adherent plates and defined media (3D, black squares). The number of clones obtained in each plate is represented in the left graph and indicated in the table to the right. Panel (**B**). Oxaliplatin sensitivity was determined by culturing the HT29 (left panel) or SW620 (right panel) cells in the presence of the indicated concentrations of oxaliplatin for 3 days. Cell survival was determined for each point and represented as percentage of survival in relation to the cell culture in the absence of the drug. Open squares represent the original cell population, black squares (Res) represent the population of cells that was made resistant to oxaliplatin, open circles represent untreated cells cultured under non-adherent (3D) conditions, and black circles represent oxaliplatin-resistant cells cultured under non-adherent conditions (3D Res). Experiments were repeated three times with similar results. Statistics: NS: non-significant differences.

Under 3D culture conditions, oxaliplatin-resistant cells were also able to form spheroids. The frequency of spheroid formation was determined and compared to the 2D condition as shown in Figure 3A. The frequency of spheroid-forming cells was higher among the resistant cells than in the initial population. In the case of HT29 cells, the frequency increased from 72.46% in the control population to 77.78% in the oxaliplatin-resistant population. This difference was more pronounced in SW620 cells, with a frequency of 13.36% in control cells versus 60% in resistant cells.

The oxaliplatin sensitivity of resistant cells cultured under 3D conditions was compared to that of resistant cells cultured in 2D conditions and with non-resistant cells cultured in both conditions. The results are shown in Figure 3B. In the two-cell lines analyzed, the lowest sensitivity (maximal resistance) corresponded to resistant cells cultured in 3D conditions (resistant CSCs). It is also interesting that 3D cultured control cells and resistant cells in 2D culture showed very similar oxaliplatin sensitivity in both cell lines.

### 3.3. Cell Invasion and Expression of Epithelial-Mesenchymal Transition Related Genes

**Figure 4:**
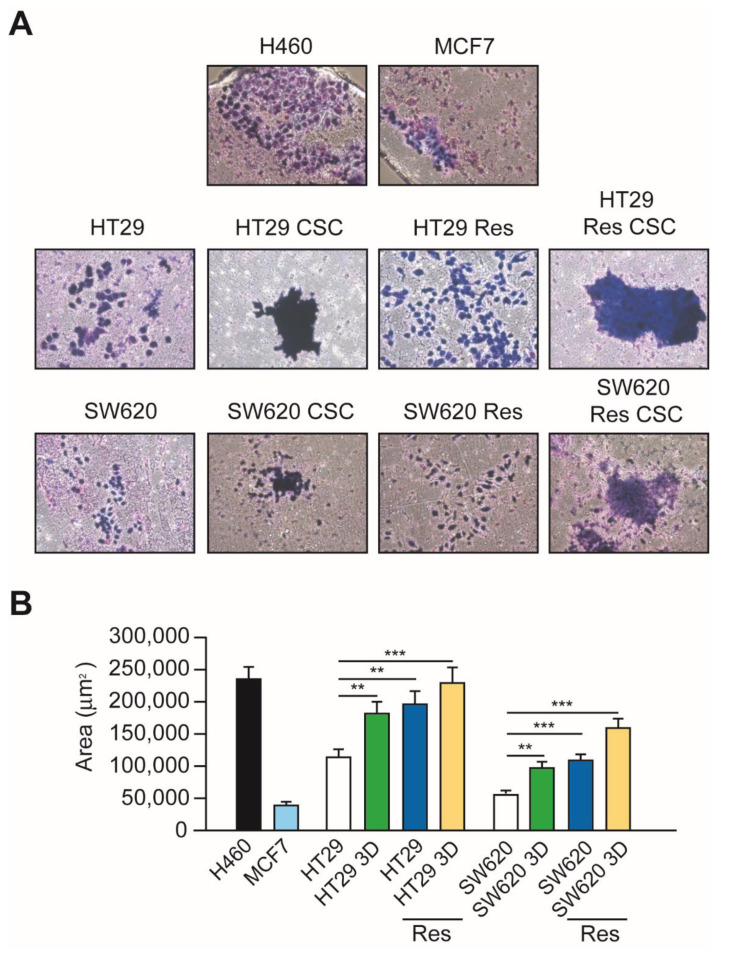
Determination of cell invasion capacity. The invasion capacity of the cells was determined using Matrigel invasion chambers and Foetal Calf Serum as a chemoattractant. HT29 and SW620 original cell lines, cells cultured under non-adherent conditions (3D), oxaliplatin-resistant cells (Res), and resistant cells grown under non-adherent conditions (Res3D) were used in these experiments. The H460 non-small cell lung cancer cell line was used as positive control and MCF7 as negative control. Panel (**A**) shows representative images of cells that migrated through the Matrigel cushion. Panel (**B**) shows the quantification of the cells that migrated (average of ten different fields). Experiments were repeated three times with similar results. Statistics: ** *p* < 0.01; *** *p* < 0.001.

Tumor progression and metastasis development are related to increased cell invasion and epithelial-mesenchymal transition. These properties were assayed in control and oxaliplatin-resistant cells cultured in either 2D or 3D conditions. Cell invasion was determined using the trans-well model. H460 cells were used as a positive control of invasive cells, whereas MCF7 cells were used as a negative control. Migrant cells were stained, and representative examples are shown in Figure 4A. Both HT29 and SW620 cells cultured under 3D conditions formed large aggregates similar to spheroids. The number of migrated cells was quantified by determining the surface of the filter covered by migrating cells (Figure 4B). The results obtained show that both cell lines grown in 3D conditions and resistant cells had an increased migration capacity in comparison to control cells, even if no morphological differences were observed among sensitive and resistant cell populations. Resistant cells grown under 3D conditions showed the largest invasion capacity in both cell lines.

**Figure 5:**
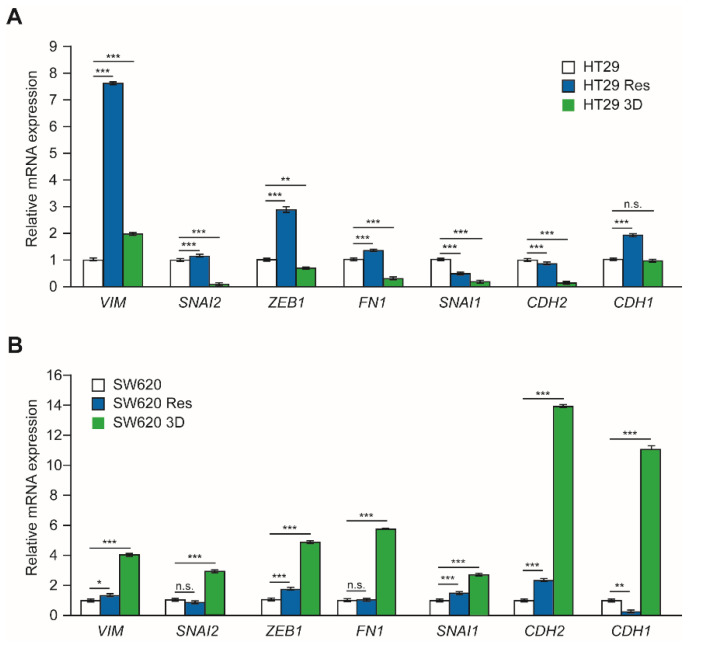
Expression of mRNAs coding for proteins involved in epithelial-mesenchymal transition (EMT). RNA was isolated from CRC cells cultured in adherent plates, non-adherent plates in defined medium (3D), and oxaliplatin-resistant cells cultured under adherent conditions (Res). The relative expression of the mRNAs coding for the protein Vimentin and the transcription factors SNAI1 (SNAIL), SNAI2 (SLUG), Zinc finger E-box-binding homeobox 1 (ZEB1), Fibronectin 1 (FN1), neural cadherin (N-cadherin), and Epithelial cadherin (E-cadherin) was determined by RT-qPCR. Panel (**A**) shows the results obtained for HT29 cells and panel (**B**) shows those obtained for SW620 cells. Experiments were repeated three times with similar results. Statistics: NS, non-significant differences; * *p* < 0.05; ** *p* < 0.01; *** *p* < 0.001.

The expression levels of mRNAs coding for several proteins involved in the epithelial-mesenchymal transition (EMT) were determined by RT-qPCR in control cells cultured in 2D and 3D conditions and oxaliplatin resistant cells (Figure 5). In HT29 cells, the expression level changes observed were larger in resistant cells, in which the EMT-related genes Vimentin (*VIM*), *ZEB1*, and *FN1* were up-regulated. Vimentin was also up-regulated in cells cultured under 3D conditions, but at a lower level (Figure 5A). Complete EMT transition is characterized by the up-regulation of N-cadherin (*CDH2*) and the down-regulation of E-cadherin (*CDH1*). However, E-cadherin was up-regulated in HT29-resistant cells, while no changes were observed in 3D cultured cells. N-cadherin levels decrease in both cell types in relation to control cells. SW620 cells cultured under 3D conditions showed a large induction of EMT-related genes (Figure 5B). In this case, the change was much reduced in resistant cells, although *ZEB1*, SNAIL (*SNAI1*), and N-cadherin showed increased expression levels in comparison to control cells. SW620 3D-cultured cells presented increased levels of E-cadherin, similar to the results previously described for HT29-resistant cells.

### 3.4. RNA Expression Analysis

**Figure 6:**
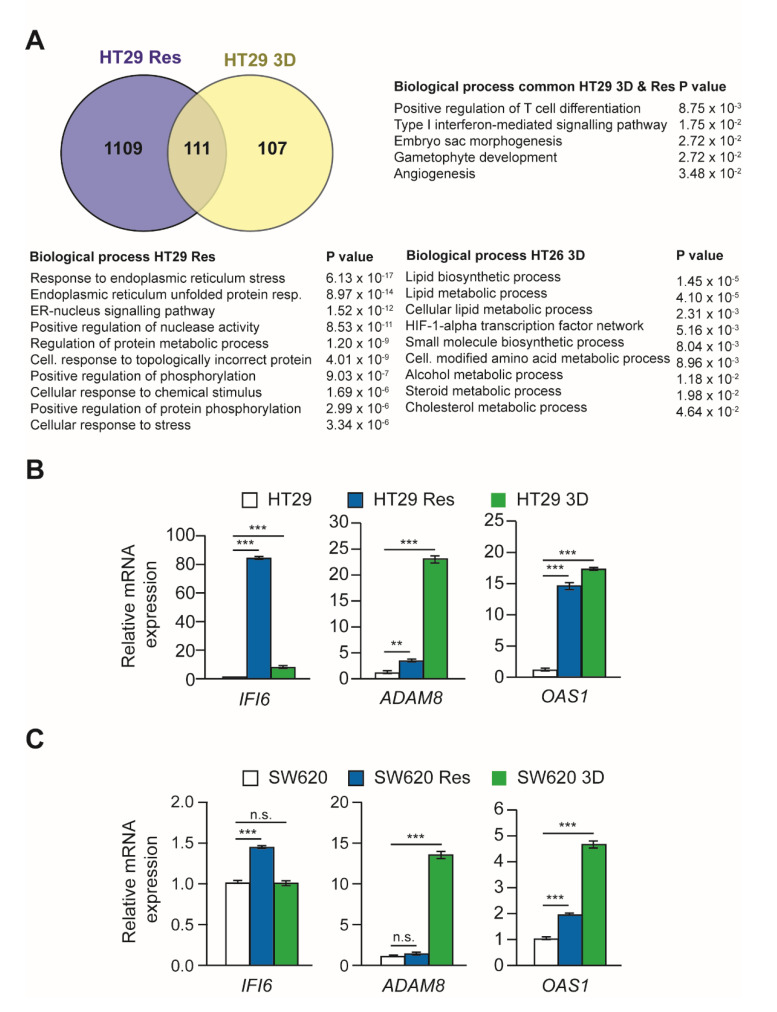
Comparative analyses of differential mRNA expression in HT29 oxaliplatin-resistant and 3D-cultured cells. Panel (**A**). mRNA was isolated from HT29 cells cultured under 2D conditions, oxaliplatin-resistant HT29 cells cultured under 2D conditions, and HT29 cells cultured under 3D conditions. Global mRNA expression levels were determined by RNA sequencing (RNAseq). Differentially expressed genes between HT29 oxaliplatin-sensitive and -resistant cells were identified (blue circle). Genes differentially expressed between HT29 cells cultured under 2D or 3D conditions were also determined (yellow circle). Finally, differentially regulated genes in these two conditions were compared and a Venn diagram of commonly regulated genes is represented. The main biological processes significantly over-represented in the genes differentially expressed in the three comparisons are shown. Panels (**B**,**C**). Comparison of the relative expression of three representative differentially expressed genes in cells cultured under 2D conditions, either sensitive or resistant (Res) to oxaliplatin, and cells cultured under 3D conditions (3D). Panel B represents the results obtained for HT29 cells, and panel C represents those obtained for SW620 cells. The mRNAs analyzed code for the proteins interferon-gamma inducible protein 6 (IFI6), ADAM Metallopeptidase Domain 8 (ADAM8), and 2′-5′-Oligoadenylate Synthetase 1 (OAS1). Experiments were repeated three times with similar results. Statistics: NS: non-significant differences; ** *p* < 0.01; *** *p* < 0.001.

The results previously described indicate a possible similarity between CSCs grown under 3D conditions and oxaliplatin-resistant cells in oxaliplatin sensitivity, invasion capacity, and EMT transition. To further search this possible relationship, an mRNA sequencing analysis was performed for HT29 cells. A comparison of the expression profile of HT29 control cells versus oxaliplatin-resistant cells identified 1219 differentially regulated genes. A similar comparison between HT29 control cells and CSCs grown under 3D conditions identified 217 differentially regulated genes. Out of these genes, 111 were commonly differentially regulated in both cell populations (Figure 6A).

The possible function of commonly regulated genes was searched through functional enrichment analysis (Figure 6A). Among the biological processes enriched were the type I interferon-mediated signaling pathway or angiogenesis, which play a pivotal role in tumor progression. The biological processes more significantly enriched in HT29 resistant cells are related to the response to endoplasmic reticulum stress. On the other hand, processes more significantly enriched in 3D-cultured cells correspond to lipid biosynthesis and metabolism and the HIF-1-alpha transcription factor network.

The differential expression of some of the genes identified in HT29 cells was confirmed by RT-qPCR analyses (Figure 6B). In addition, their possible regulation in SW620 cells was also studied (Figure 6C). The results obtained indicated that the three genes analyzed (*IFI6*, *ADAM8*, and *OAS1*) were induced both in HT29 resistant and 3D cultured cells to different degrees. The *OAS1* gene was also up-regulated in SW620 oxaliplatin-resistant and 3D-cultured cells, while *ADAM8* was highly induced in SW620 3D cultured cells, and IFI6 was up-regulated in SW620 oxaliplatin-resistant cells.

## 4. Discussion

Tumor regression is one of the main hindrances for CRC treatment and patient survival. One of the possible causes is the induction of drug resistance through different mechanisms. Another is the persistence of a population of cancer stem cells that is not sensitive to the chemotherapeutic drug and can regenerate the tumor. In this article, a cell culture model was established to study these two aspects of tumor progression and their possible interactions. Colorectal CSCs were obtained by cell culture under non-adherent conditions in defined, serum-free media as previously described [31,36,39]. Oxaliplatin-resistant cells were induced by a single treatment with a drug concentration corresponding to the IC80 concentration for each of the two cell lines analyzed. This protocol of single-dose treatment was intended to mimic clinical treatments based on single doses periodically administered. Previous studies induced resistance by continuous incubation of the cells with sub-lethal drugs concentrations [29,40]. The data obtained showed several correlations between these two cell populations. One of them is that CSCs showed lower sensitivity to oxaliplatin than the same cells cultured under 2D conditions. Actually, the sensitivity of SW620 cells was significantly higher than that of HT29 cells (Figure 1D and Figure 2E). However, the reduced proportion of CSCs isolated from SW620 cells showed an oxaliplatin sensitivity similar to that of HT29 CSCs (Figure 1D). In addition, oxaliplatin-resistant cells showed higher efficiency of CSC spheroid formation than the original cell populations. This increase was larger in the cell line more sensitive to oxaliplatin, SW620 (Figure 2), as also observed for CSCs. Furthermore, CSCs derived from resistant cells showed the lowest sensitivity and, therefore, the highest drug resistance of the cell populations analyzed, indicating possible cooperation of both cellular stages.

The similar behavior of CSCs and resistant CRC cells extended to other characteristics. Both cell types showed increased invasion capacity, regardless of whether they were derived from HT29 or SW620 cells. The main difference is that CSCs formed large aggregates and resistant cells did not, suggesting that they may use collective migration instead of individual migration. CSCs derived from resistant cells showed the highest invasion capacity among the cell types analyzed and formed large aggregates. The formation of large aggregates after cell invasion by CSCs has been previously observed by Han et al. [39] and in non-small cell lung cancer [31]. Resistant and CSCs also presented increased expression of several EMT-related genes. In the case of HT29 cells, EMT induction was mainly observed in resistant cells, although Vimentin expression was also induced in CSCs. On the contrary, EMT was more pronounced in SW620-derived CSCs. Some of the EMT-related genes analyzed, such as SLUG, SNAIL, and N-cadherin were not induced in HT29-resistant cells or CSCs. E-cadherin expression was not repressed in HT29-derived cells either. Similarly, E-cadherin expression was not repressed in SW620 CSCs. Similar data on E-cadherin expression were previously described for HT29 cells grown under 3D culture conditions [36,41]. These data might be indicative of a partial EMT, as previously described in other cancer models.

Transcriptomic analyses were designed to obtain more general information on the possible similarities and differences between oxaliplatin-resistant and CSCs derived from HT29 cells. The results indicate that out of 217 genes differentially regulated in CSCs in relation to the original population, 111 (51%) were also differentially regulated in resistant cells. This high coincidence might indicate that many of the transcriptional changes associated with the induction of CSCs also occur in resistant cells. These data are in agreement with the functional data showing the similarities in oxaliplatin sensitivity, cell invasion, and EMT discussed previously. The number of genes differentially regulated in oxaliplatin-resistant cells was much higher (1219), indicating that the induction of drug resistance might involve other regulatory and genetic pathways that are not changed in CSCs in comparison to cells grown under 2D conditions. In the case of SW620 cells, only three genes have been analyzed that partially coincide with the expression levels in HT29 cells. However, a complete transcriptomic analysis of SW620-derived cells and of other CRC cell lines would be necessary to confirm these results and to perform more comprehensive analyses of the gene expression mechanisms that regulate CSCs and oxaliplatin resistance in CRC.

Previous studies have described that induced CRC CSCs present increased resistance to chemotherapeutic drugs, including oxaliplatin. Kawamoto et al. isolated SW620 CSCs that expressed CD133 and showed increased resistance to irradiation and 5-fluorouracil [42]. Induction of EMT by Snail overexpression induced a CSC-like phenotype in HT29 CRC cells and increased resistance to oxaliplatin [26]. In SW620 cells, the inhibition of SNAI1 expression decreased spheroid formation and radiation resistance [27]. Syntenin-1 knockdown in SW620 cells reduced the presence of CSCs, oxaliplatin chemoresistance, and cell migration [43].

In addition, chemotherapy-resistant cells have been described to increase CSCs characteristics. For example, 5-fluorouracil treatment of SW403, HCT116, and SW620 cells increased the fraction of cells expressing ALDH, which was used as a CSC marker in this study [44]. HT29 cells made resistant to oxaliplatin by continuous exposure to the drug showed 30-fold enrichment of CD133^+^ cells [29]. CRC samples cultured as mice xenografts also showed that chemotherapy treatment increased the presence of a cell population with CSC characteristics. Additionally, the increase of the CSC population by ZEB2 overexpression increased chemoresistance in the CRC cells of the xenografts [45].

Huang et al. published one study related to the one presented here using the HCT116 CRC cell line [46]. These authors isolated CRC CSCs by culture in a defined medium under non-adherent conditions. Oxaliplatin- and 5-fluorouracil-resistant cells lines were generated by long-term culture, in contrast to the method used in the present study. Huang et al. described that CSCs and resistant cells were enriched in CSC markers and presented increased resistance to the drugs. Resistant cells also showed increased clonogenic capacity. On the other hand, the phenotypic similarity between cisplatin-resistant and CSCs in non-small cell lung cancer cells was previously described by our group [31].

Cancer stem cells have been considered to possibly originate from the malignant transformation of tissue stem cells. However, other studies have proposed that differentiated cancer cells can also become cancer stem cells. Ohata et al. described the conversion of differentiated CRC cells expressing a low amount of the CD44 stem cell marker into high CD44 expressing cells [47]. Feng et al. characterized the conversion of SW620 CD133^−^ cells into CD133^+^ CSCs [25]. The existence of a dynamic equilibrium between SW620 CSC and non-CSC populations has been proposed by several authors [48,49,50]. Both models of CSC generation could explain the results obtained in this study. The first possibility is that both cell lines initially contain a population of CSCs that can form spheroids under 3D culture conditions. The proportion of these cells would be larger in HT29 cells than in SW620 cells. These cells would be the main components of the oxaliplatin-resistant population, so these resistant cells would form spheroids at a higher frequency than the initial population. Alternatively, culture under 3D conditions could induce the transition of a number of cells to the CSC stage, and this induction would be more frequent in HT29 than in SW620 cells. Oxaliplatin treatment would induce drug resistance mechanisms in the cells, and these changes could favor the transition to CSCs identified under 3D culture conditions. This second scenario could have clinical implications because it would predict that the development of drug resistance in the patients could also increase the number of CSCs, and these cells could also be resistant to other drugs. This situation would strengthen the importance of the development of CSC-efficient drugs for the treatment of resistant tumors. The confirmation or rejection of these hypotheses requires further work in the characterization of the cellular components of cancer cell lines and primary tumors.

## 5. Conclusions

The data presented using two different cell lines indicate that colorectal cancer stem cells and oxaliplatin-resistant cells are very related cell populations. Cancer stem cells presented increased oxaliplatin resistance, while resistant cells contained a larger proportion of stem cells than the original cell populations. In addition, stem cells isolated from oxaliplatin-resistant cells showed the highest resistance to oxaliplatin among the studied cell populations. Both cancer stem cells and oxaliplatin-resistant cells also had increased cell invasion capacity and regulated the expression of some epithelial-mesenchymal transition-related genes similarly. Transcriptome analysis indicated that a relevant proportion of genes presented a similar differential expression in cancer stem cells and oxaliplatin-resistant cells.

## Data Availability

Data are available from the corresponding authors upon request.
